# Time-Dependent Degradation of Polyphenols from Thermally-Processed Berries and Their In Vitro Antiproliferative Effects against Melanoma

**DOI:** 10.3390/molecules23102534

**Published:** 2018-10-04

**Authors:** Zorița Diaconeasa

**Affiliations:** Faculty of Food Science and Technology, University of Agricultural Science and Veterinary Medicine Cluj-Napoca, Calea Mănăştur 3-5, 400372 Cluj-Napoca, Romania; zorita.sconta@usamvcluj.ro; Tel.: +40-751-033-871

**Keywords:** berries jam, phenolic acids, flavonols glycosides, degradation, HPLC-ESI/MS

## Abstract

Polyphenols are natural occurring micronutrients that can protect plants from natural weathering and are also helpful to humans. These compounds are abundantly found in fruits or berries. Because of berry seasonal availability and also due to their rapid degradation, people have found multiple ways to preserve them. The most common options are freezing or making jams. Polyphenol stability, during processing is a continuous challenge for the food industry. There are also multiple published data providing that they are sensitive to light, pH or high temperature, vectors which are all present during jam preparation. In this context the aim of this study was to assess phytochemical composition and bioactive compounds degradation after jam preparation. We also monitored their degradation during storage time and their in vitro antiproliferative potential when tested on melanoma cells. The obtained results revealed that when processed and stored in time, the bioactive compounds from berries jams are degrading, but they still exert antioxidant and antiproliferative potential. Prior to LC-MS analysis, polyphenolic compounds were identified as: flavonoids (anthocyanins (ANT), flavonols (FLA)) and non-flavonoid (hydroxycinnamic acids (HCA) and hydroxybenzoic acids (HBA)). The most significant decrease was observed for HCA compared to other classes of compounds. This variation is expected due to differences in constituents and phenolic types among different analyzed berries.

## 1. Introduction

Fruits and vegetables are important sources of bioactive compounds which were shown to have positive health benefits [1,2]. In recent years, food and nutrition sciences aimed to improve health through intelligent foods containing bioactive plant-based molecules that were proven to have positive health benefits, such as the prevention of cardiovascular diseases or cancer [3,4]. The demand for innovative and functional food products had increased lately due to the fact that consumers are more aware of their bodies and mental health. Diets rich in vegetables or fruits were proven to provide essential bioactive molecules which can play important roles in human health. These plant-based bioactive molecules include polyphenols (phenolic acids, flavonoids, anthocyanins, catechins), enzymes, amino acids, vitamins (vitamin C, folate, and provitamin A), minerals (potassium, calcium, and magnesium), and fibers (inulin, pectin, lignan). The related body of literature indicates a strong correlation between diet and degenerative diseases, and due to this fact, the use of natural compounds as ingredients in food has become a major concern for food technologists [5,6]. Berries and fruits are the main sources of bioactive compounds with many applications in the food, pharmaceutical, nutraceutical and cosmetic industries [7,8,9,10]. These bioactive compounds are mainly polyphenols, and among them, anthocyanins which have proved to have high nutritional and potential health value [11]. In vivo studies have shown that anthocyanins have many positive effects on the prevention of cardiovascular diseases, diabetes, and cancers (lung, colon, breast, and skin). Red berries, including the chokeberry, blueberry, blackcurrant, elderberry, raspberry, and cranberry, are widely consumed fresh or in processed forms, such as jams, juices, syrups, and various types of jellies. These fruits have been extensively investigated from the chemical point of view, particularly in fresh, juice, or dried forms [12]. Due to the fact that fresh berry consumption is not always possible, jam production is a good option for the food industry. In order to obtain high quality berry jams, the technological process must use low temperatures, environmentally-friendly and non-destructive methods. In this way, bioactive compounds can be protected from degradation, and the colour of the products can also be preserved. In order to influence consumers’ acceptance, the product—jam in this case—must still have an attractive colour. This colour is related to the anthocyanin content and has a strong association with the antioxidant capacity. However, the temperature and time of processing must be chosen properly to ensure the stability of the anthocyanins and the conservation of the antioxidant activity [13,14,15]. Moreover, several other factors can affect the colour of berry jams, including the storage temperature, amount of light exposure, and pH [16,17,18]. The antioxidant capacity can decrease, remain unchanged, or even increase during processing or storage. Previous studies have revealed that the optimum storage temperature for jam is 4 °C, and the brief storage of raspberry jam at 4 °C has been associated with a lower rate of anthocyanin degradation compared to jam stored at a higher temperature (15 °C) [19]. The thermal treatment of anthocyanins is related to both anthocyanin and antioxidant capacity degradation [20]. In this context, the aim of present study is to prepare homemade jams using chokeberry, elderberry, blackcurrant, or blackthorn and to evaluate phenolic compounds degradation during storage time. Moreover the in vitro antiproliferative potential of the rich polyphenolic extracts will be tested on melanoma cell line. However, to the best of our knowledge, no previous studies have investigated the effect of food compounds processing on the phenolic compound content and the antioxidant capacity of homemade jams prepared using chokeberry (*Aronia melanocarpa*), elderberry (*Sambucus nigra*), blackcurrant (*Ribes nigrum*) or blackthorn (*Prunus spinosa*).

## 2. Results

### 2.1. LC-PDA-ESI/MS Identification and Quantification of Phenolic Compounds

#### 2.1.1. Chokeberry Jam

**Figure 1:**
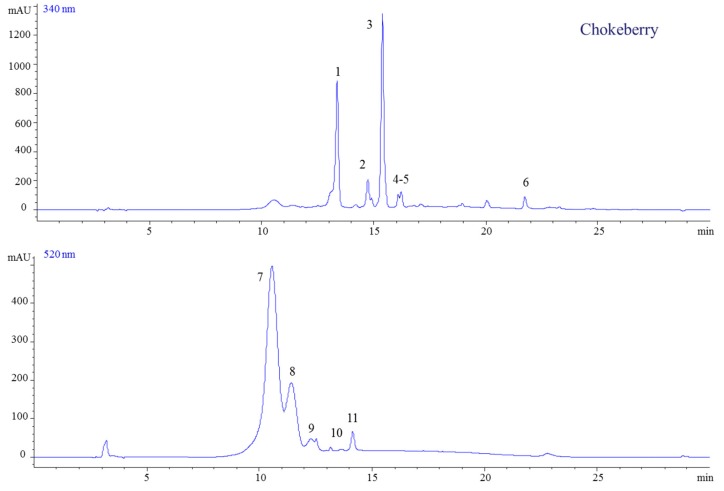
HPLC-DAD chromatogram of extracts from chokeberry jams (at 340 and 520 nm).

**Figure 2:**
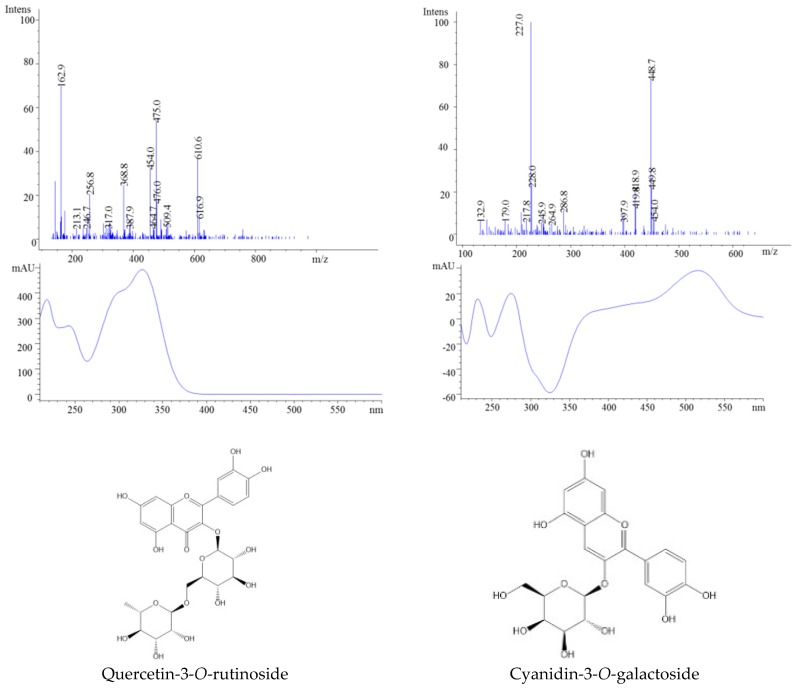
HPLC-MS spectra, UV/vis scanning spectra, and the chemical structures of peaks 3 and 7.

The obtained jams and fresh berries were characterized by the presence of 11 compounds: five anthocyanins, four flavonols, one HCA and one HBA (Table 1). The identified anthocyanins were only glycosylated cyanidin, whereas, in the case of flavonols, we identified glycosylated quercetin as well as caffeic and ellagic acids which are forms of HCA and HBA, respectively. Cyanidin-3-*O*-galactoside was found to be the main compound among all the identified anthocyanins (Figure 1). It was identified by the *m*/*z* 449 molecular ion, which was confirmed by the fragment ion *m*/*z* 287, which corresponds to aglycone cyanidin (Figure 2).

The ESI-MS analysis of peaks 8, 9, and 10 showed the presence of molecular ions at *m*/*z* 419, corresponding to cyanidin-3-*O*-arabinoside; *m*/*z* 449, corresponding to cyanidin-3-*O*-glucoside; and *m*/*z* 419, corresponding to cyanidin-3-*O*-xyloside. Regarding the quantitative analysis, the flavonol profiles were consistent with those reported previously, except that quercetin-3-*O*-rutinoside was found to be present in a higher amount [21,22]. In addition to the flavonols identified in our study, Mikulic-Petkovsek et al. identified four more quercetin glycosides: glucuronide, xyloside, arabinopyranoside, and robinobioside [21,23]. At peak 1, the MS parent ion *m*/*z* 163 was identified as caffeic acid which is in agreement with the available literature [24].

**Figure 3:**
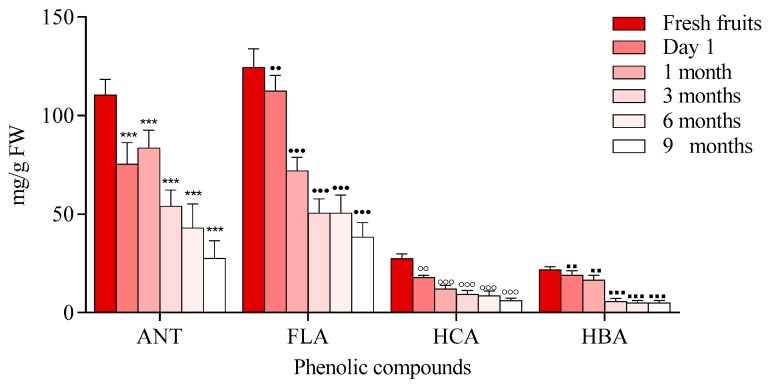
Phenolic compound degradation of chokeberry jams during storage, (anthocyanins (ANT), flavonols (FLA), (hydroxycinnamic acids (HCA) hydroxybenzoic acids (HBA)) Data represents the means ± SEM of at least three independent experiments (significant differences, **^, ●●, ◦◦, ▪▪^ *p* < 0.01, ***^, ●●●, ◦◦◦, ▪▪▪^ *p* < 0.001).

A decrease in the content of anthocyanins was observed immediately after processing and also, during storage time as it is shown in Figure 3. 

This degradation can be attributed to the hydrolytic reactions that led to the conversion of anthocyanin glycosides into chalcones, whose form can be rapidly transformed into phenolic acids and aldehydes.

Moreover, heat stable forms of polyphenol oxidase or peroxidase may play roles in the reduction of anthocyanins [30,31]. Another mechanism that may be associated with anthocyanin degradation is related to the hydrolysis of the glycoside linkages. This hydrolysis is known as the first step towards anthocyanin degradation, because high temperatures can shift the anthocyanin equilibrium towards the colorless chalcones. Chalcone degradation can occur due to the presence of oxidation reactions which can generate brown compounds or pigments that have high molecular weights. Additionally, pH values can affect the flavylium salt degradation, which is stable under highly acidic conditions. At higher pH values, salts can lose a proton and are easily transformed into an unstable pigment (quinoidal base) that is bonded to water and forms a colorless compound, commonly known as chromanol [30,32]. During processing and at the end of the storage period, anthocyanin degradation ranged from 58.90% to 74.30%. The results of the loss of anthocyanin content were lower compared with data reported previously for processed and stored black carrot jam and marmalade (87.60–95.60%). Degradation was also observed in flavonoids immediately after processing and during storage. This finding is in agreement with the previously reported data for *Rubus coreanus* Miquel berry jams [33]. The results revealed that jams prepared at pH 2.0–3.0 lost 33–35% of their anthocyanins, while the total amount of anthocyanins in jams obtained at pH 3.5–4.0 were degraded by 40–48%. In another study on blueberry jam, in contrast to other polyphenolics, the level of total flavonols was stable in response to processing, with >94% retention, compared to levels found in fresh berries [31] (Figure 3). Our study showed a degradation of HBA compound ranging from 70 to 90%. This had the highest degradation rate comparing with other existing compounds. 

Chokeberries are a rich source of anthocyanins compared with other fruits. Due to this, they are usually used in the food industry as colorants or as a supplementary source of antioxidants. A recent study showed that the supplementation of strawberry jams with chokeberries and flowering quince during processing increase in the content of phenolic components in final products, especially for proanthocyanidins [34]. 

#### 2.1.2. Blackthorn 

**Figure 4:**
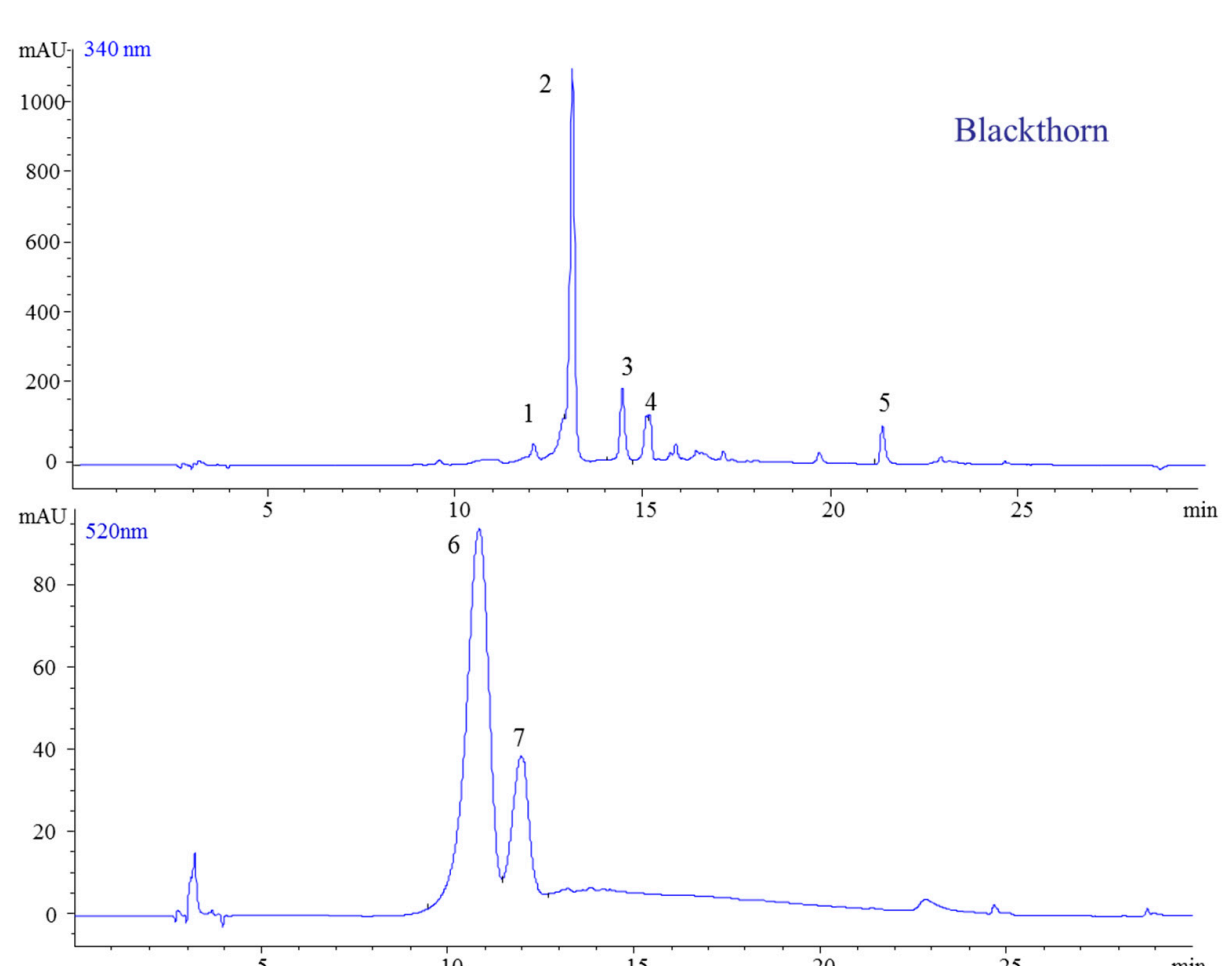
HPLC-DAD chromatogram of extracts from blackthorn jams (at 340 and 520 nm).

These berries are becoming very popular in the food industry due to their complex and valuable phytochemical composition and also due to their easily availability in nature. The chromatographic analysis of fresh berries or jams revealed the presence of five phenolic compounds (Figure 4). 

The identified compounds are summarized in Table 1. The mass spectra of peak 1 displayed a parent ion at *m*/*z* 181 and one fragment ion at *m*/*z* 163, which was identified as caffeic acid, while peak 2 had an ion at *m*/*z* 181 which was identified as neochlorogenic acid. Two more molecules were identified as quercetin (*m*/*z* 303) and quercetin-3-*O*-rutinoside (*m*/*z* 611). Anthocyanins were shown to be the most abundant class (fresh berries, jam), followed by flavonols and HCA. In contrast to the other berries in this study, blackthorn did not contain HBA. The identified anthocyanins were two glycosylated cyanidins and one acylated peonidin. For peak 5, in the case of cyanidins, the ESI-MS analysis indicated the presence of a molecular ion at *m*/*z* 449 corresponding to cyanidin-3-*O*-galactoside. 

**Figure 5:**
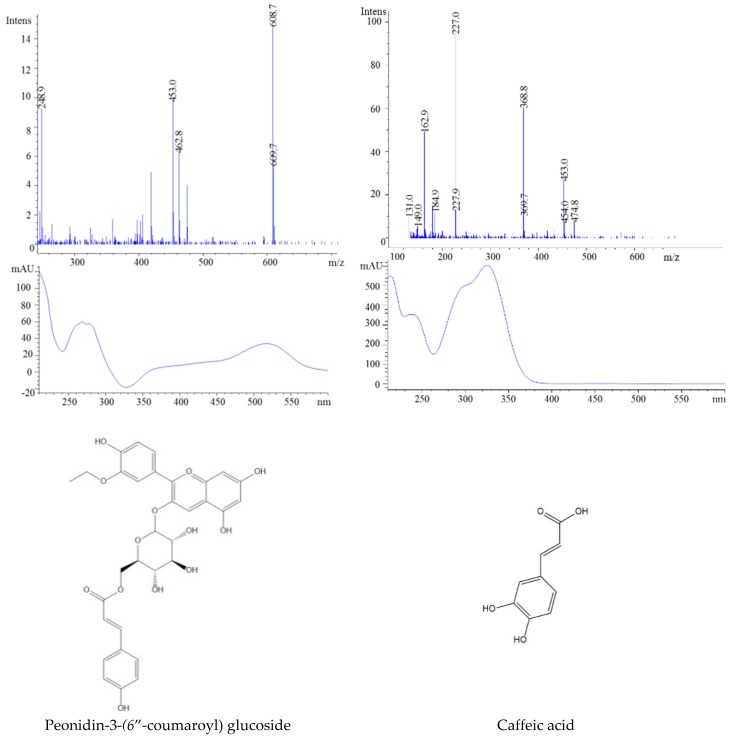
HPLC-MS spectra, UV/vis scanning spectra, and the chemical structures of peaks 1 and 7.

The second isolated anthocyanin showed a molecular ion at *m*/*z* 595, suggesting the presence of cyanidin-3-*O*-rutinoside. In this case, the ion at *m*/*z* 449 showed a loss of one molecule of rhamnoside and an ion at *m*/*z* 287, confirming the presence of aglycone cyanidin. Further, the peaks, registered with a molecular ion at *m*/*z* 603 and a fragment ion at *m*/*z* 301, were identified as peonidin 3-(6″-coumaroyl) glucoside. Peak 4 had a molecular ion at *m*/*z* 609 and two fragment ions at *m*/*z* 463 and *m*/*z* 301, which indicated the presence of peonidin 3-(6″-coumaroyl) glucoside (Figure 5). This anthocyanin identification is in agreement with Stefǎnuț et al., who also reported the presence of peonidin-3-*O*-rutinoside [26]. These differences may be attributed to the climatic conditions or harvesting time.

**Figure 6:**
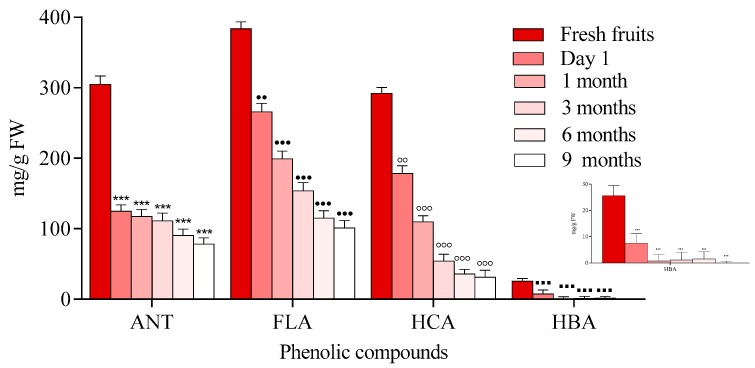
Phenolic compounds degradation in blackthorn jam during storage, (anthocyanins (ANT), flavonols (FLA), (hydroxycinnamic acids (HCA) hydroxybenzoic acids (HBA)). Data represents the means ± SEM of at least three independent experiments (significant differences, **^, ●●, ◦◦, ▪▪^ *p* < 0.01, ***^, ●●●, ◦◦◦, ▪▪▪^ *p* < 0.001).

After processing, the bioactive compounds of blackthorn jam decreased (Figure 6). The amount of anthocyanins in berries decreased by 50% immediately after thermal processing compared to fresh ones and reached 82.56% after 6 months of storage. The same results were reported for black carrot (*Daucus carota*) jams and marmalades [30]. After 20 weeks of storage, the preserved anthocyanins and antioxidant capacity in samples stored at 4 °C were 53.4–81.0% and 45.2–92.0%, respectively. Time-dependent degradation of flavonols, ranging from 23 to 67%, was also observed. As in the case of HCA, the amounts of caffeic and neochlorogenic acid dramatically decreased from 41 to 81% during storage time.

#### 2.1.3. Elderberry

**Figure 7:**
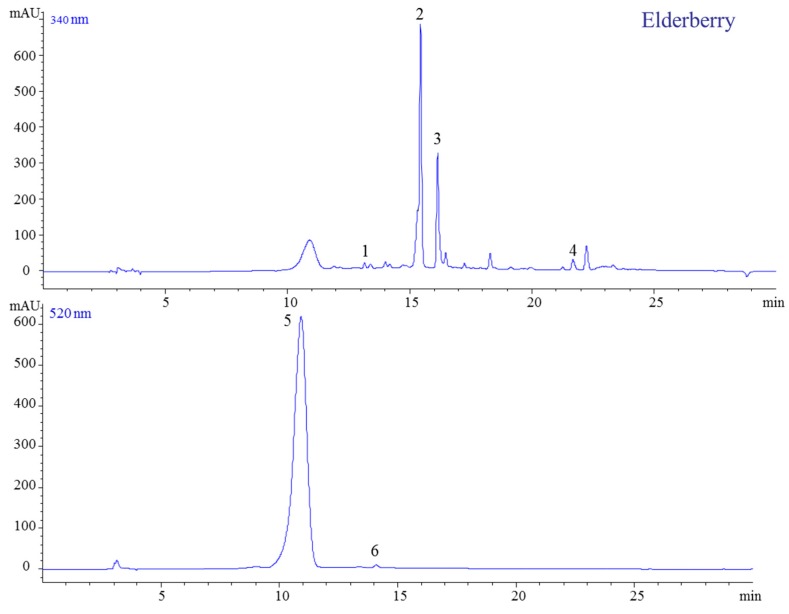
HPLC-DAD chromatogram of berry extracts from elderberry jams (340 and 520 nm).

Thus far, elderberries were not very popular for consumers due to their alkaloid content. However, this can easily be neutralized by thermal processing. Recently, an interest in elderberries has developed due to their rich polyphenolic compound content and thus, high antioxidant potential. Consequently, these berries are now becoming a very popular crop in Europe, and thus, we included them in our study. The HPLC analysis revealed that elderberry has a simple phenolic fingerprint, characterized by the presence of six compounds: two anthocyanins and four hydroxycinnamic acids (HCA) (Figure 7). 

**Figure 8:**
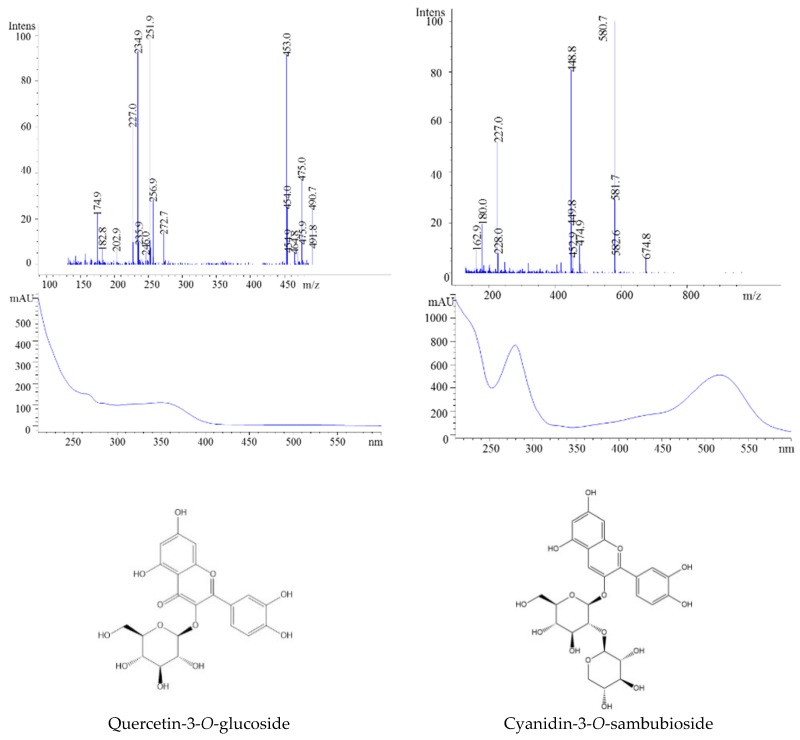
HPLC-MS spectra, UV/vis scanning spectra, and the chemical structures of peaks 2 and 5.

The identified anthocyanins in fresh fruits and prepared jam were exclusively cyanidin-based anthocyanins, quercetin derivatives, and chlorogenic acid. Peak 1 was assigned to chlorogenic acid because it has a parent ion at *m*/*z* 355 and two fragments ions at *m*/*z* 181 and 163. The next three peaks (2, 3, 4) were found to be quercetin-3-*O*-rutinoside (*m*/*z* 611, 303), quercetin-3-*O*-glucoside (465, 303), and quercetin (303) (Figure 8). Further, the ESI-MS analysis showed the presence of molecular ions at *m*/*z* 381 corresponding to cyanidin-3-*O*-sambubioside, while peak 6 was identified as cyanidin-3-*O*-glucoside (*m*/*z* 449, 287) (Table 1, Figure 8). 

**Figure 9:**
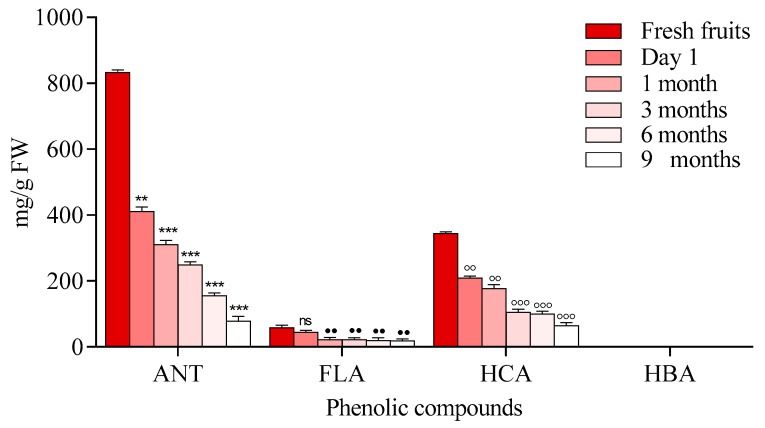
Phenolic compound degradation in elderberry jam during storage, (anthocyanins (ANT), flavonols (FLA), (hydroxycinnamic acids (HCA) hydroxybenzoic acids (HBA)). Data represents the means ± SEM of at least three independent experiments (significant differences, **^, ●●, ◦◦, ▪▪^ *p* < 0.01, ***^, ●●●, ◦◦◦, ▪▪▪^ *p* < 0.001).

Three flavonols were identified in this sample, with quercetin-3-*O*-rutinoside (Figure 7) being the major one, followed by quercetin-3-*O*-glucoside. This rank was maintained after thermal processing and storage (Figure 9).

The obtained results regarding phenolic changes during processing are in agreement with the available literature [35]. The current body of literature indicates that thermal processes have a large influence on flavonoid stability, especially for rutin which has higher stability compared to its aglycon form (quercetin) [32,33]. These findings were attributed to the presence of carbanion formation because of the glycosylation of the 3-hydroxyl group in the C-ring. 

Other authors also reported that glycosylated form are more stable: luteolin was found more resistant to heat than rutin or luteolin-7-glucoside when heated at 180 °C for 180 min [34]. Moreover, the highest level of polyphenolic compounds was observed in the extraction of *Orthosiphon stanmineus* leaf, with 80% methanol at 40 °C and a significant degradation of the analytes recorded at temperatures above 60 °C [16]. The results of the previously cited study also showed a significant reduction in the free radical-scavenging activity of the samples which were treated at temperatures above 60 °C. To conclude, the antioxidant capacity of the flavonoids, and therefore, their pathway to oxidative degradation is linked with their special structural features. 

#### 2.1.4. The Blackcurrant

**Figure 10:**
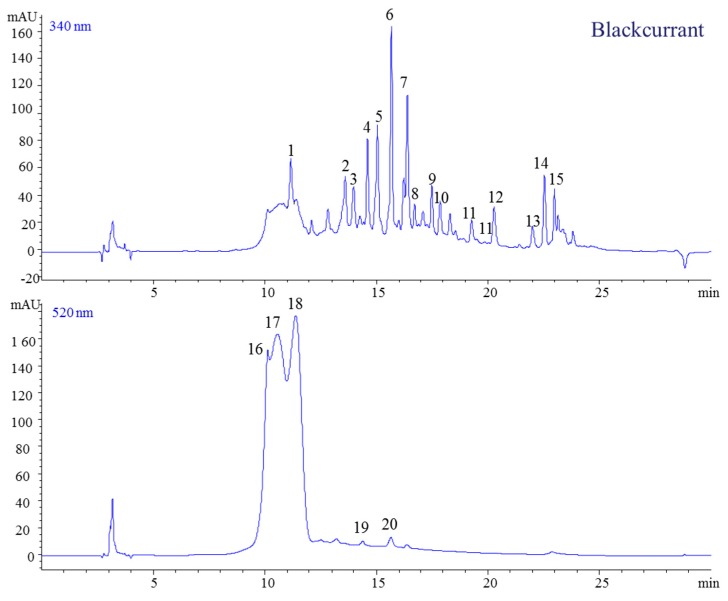
Chromatographic profile of berry extracts from blackcurrant jams (340 and 520 nm).

This sample had the most complex matrix of polyphenol constituents. Prior to the ESI-MS analysis, we were able to identify 20 individual compounds (Figure 10). The available literature data indicates that the polyphenolic compounds present in blackcurrant are caffeic acid, hydroxybenzoic acid, quercetin, myricetin, cyanidin, and delphinidin [28,29,36]. Table 1 presents the retention times, molecular ions, and fragmentation information for all polyphenolic compounds found in the present study. The mass spectra of peak 1 displayed a parent ion at *m*/*z* 341 and one fragment ion at *m*/*z* 139 which was identified as 4-hydroxybenzoic acid-4-*O*-glucoside. This sample was also characterized by the presence of 13 flavonols-mainly kaempferol-as well as quercetin or myricetin derivates. For anthocyanins, glucosides and rutinosides were the main sugar moieties of delphinidin and cyanidin identified (Table 1). Overall, the anthocyanins identified in the present study are in agreement with those detected in previous studies [12].

**Figure 11:**
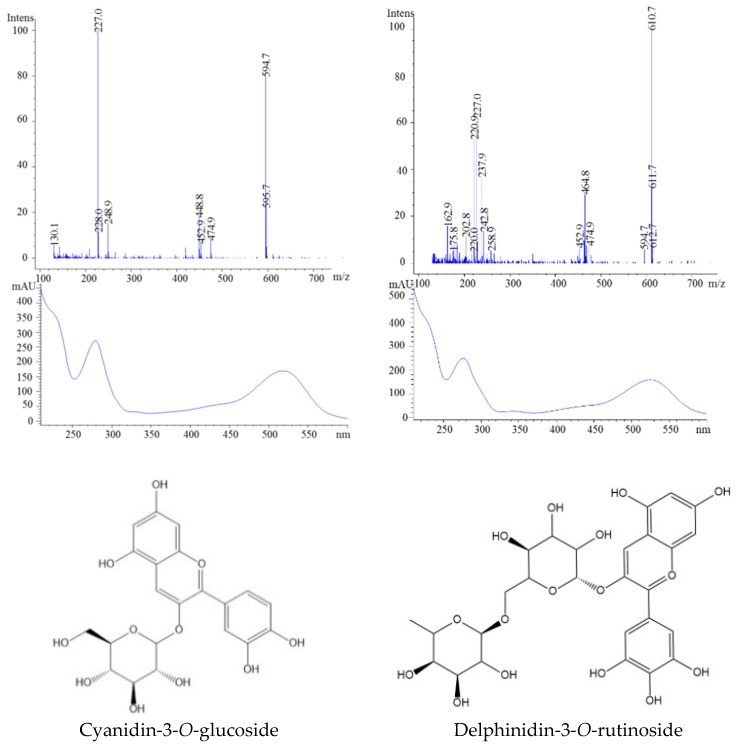
HPLC-MS spectra, UV/vis scanning spectra, and the chemical structures of peaks 17 and 18.

The first identified anthocyanin (peak 1) was delphinidin-3-*O*-glucoside (*m*/*z* 303), while the second isolated anthocyanin showed a parent ion at 611 and a molecular ion at *m*/*z*, suggesting the presence of delphinidin-3-*O*-rutinoside (Figure 11). Moreover, the ion at *m*/*z* 449 indicated the loss of one molecule of rhamnoside, and the ion at *m*/*z* 287 confirmed the presence of aglycone cyanidin (Table 1). 

**Figure 12:**
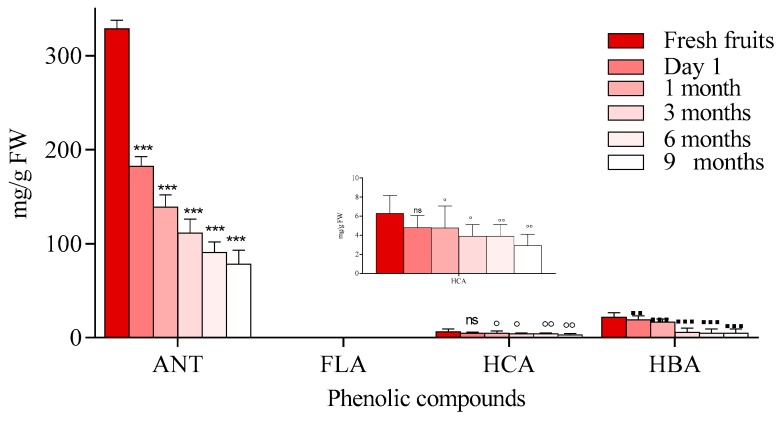
Phenolic compound degradation in blackcurrant jam during storage (anthocyanins (ANT), flavonols (FLA), (hydroxycinnamic acids (HCA) hydroxybenzoic acids (HBA)). Data represents the means ± SEM of at least three independent experiments (significant differences, *^, ●, °, ▪^ *p* < 0.05, **^, ●●, ◦◦, ▪▪^ *p* < 0.01, ***^, ●●●, ◦◦◦, ▪▪▪^ *p* < 0.001).

Our results indicated that heating and prolonged storage time are influencing the contents of the individual phenolic compounds, and moreover, their content decreased overall (Figure 12). These findings are in agreement with a previous study [37] which reported that flavonoid loss may depend on the preparation method used, such as boiling, frying with oil and butter, or microwaving. 

### 2.2. Cell Proliferation

**Figure 13:**
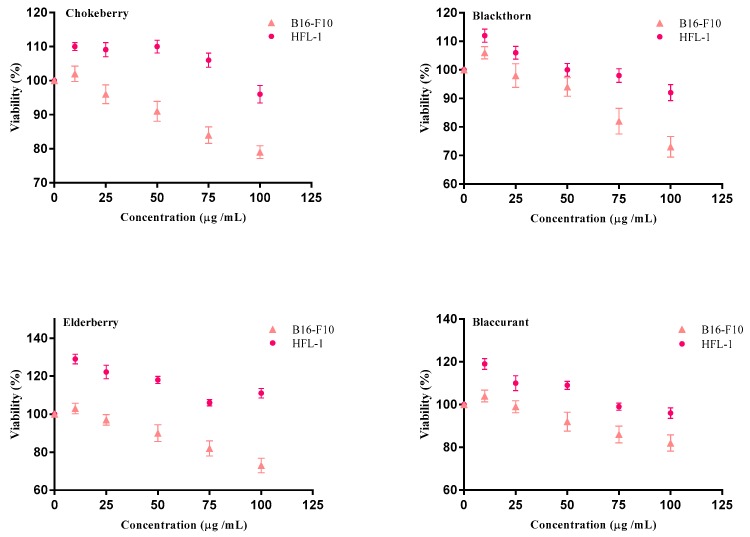
Effect of RPE from chokeberry, blackthorn, elderberry and blackcurrant on B16-F10 and HFL-1 cell proliferation.

Cell proliferation was performed by evaluating the mitochondrial succinate dehydrogenase activity of both cell lines (normal and melanoma) after applying a 24 h treatment with RPE. In the case of a normal fibroblast cell line (HFL-1), all the extracts showed a stimulation of proliferation, especially elderberry extract, while blackthorn extract exerted a less significant influence on cell proliferation. These differences could be explained by their varied phytochemical compositions. In this context we can state that elderberry extract was characterised by the presence of anthocyanins and HCA, while blackthorn, in addition to its anthocyanin and HCA contents, was shown to contain flavonols as well. Further, for the human melanoma cell line (A375), the applied treatments reduced cell proliferation after 24 h at the highest applied concentration (100 μg/mL) (Figure 13). The obtained results were well correlated with the dose concentration. Moreover, data showed that all of the applied treatments on the melanoma cell line have a cell proliferation effect ranging from 20–25%. The extract with the highest antiproliferative potential was the one obtained from blackthorn. This fact can be attributed to their phytochemical composition which is abundant in glycosylated and acylated anthocyanins (356 mg/100 g FW). In other words, there was no dose that inhibited 50% of the cultivated cells. The obtained results demonstrate that extracts rich in polyphenols have antiproliferative potential on tumour cells, while on normal cells, they have been proven to stimulate cell proliferation in a dose dependent manner. 

This fact is in agreement with other published data [38]. However, like all natural plant metabolites, anthocyanins are unstable and highly susceptible to degradation. Their stability is highly influenced by pH, temperature, light and also the presence of complexing compounds such as other phenolic acid and flavonoids or metals ions [39]. Moreover, it is not clear how exactly anthocyanins act at cellular level, but their proprieties seem to be closely related to their antioxidant activity [40]. Being unstable molecules, when exposed to high pH values, during the in vitro testing, anthocyanins can rapidly degrade or be breakdown in different metabolites. Very little is known about the details and the mechanisms of anthocyanin absorption and transportation when comparing with other flavonoid groups, such as flavonols. Anthocyanins exhibit complex biochemistry and much remained to be discovered about the biochemical activity of these compounds.

Currently, most investigations on anthocyanins are focusing on solving these problems, as well as anthocyanin bioavailability which seems to be very low with <1% absorption from the ingested dietary dose [41]. Moreover anthocyanins are subject to degradation in vivo, resulting in a breakdown to phenolic acids and aldehydes [42], such as protocatechuic acid (PCA) and phloroglucinol aldehyde (PGA) in the case of cyanidin. For other anthocyanins classes such as glycosylated or acylated form, the consumption and bioavailability of dietary phenolics have become a major concern in phenolic chemopreventive and cancer therapy research and these were not researched as far as we know.

## 3. Discussion

The obtained results revealed that during processing and storage, the bioactive compounds from all berry jams degraded over time, but they still exert antioxidant activity. Prior to the chromatographic analysis, polyphenolic compounds were identified as flavonoids (anthocyanins (ANT), flavonols (FLA)) or non-flavonoids (phenolic acids derivates of hydroxycinnamic acids (HCA) and hydroxybenzoic acids (HBA)). The polyphenolic compounds were identified by comparing the peak *m*/*z* of each molecule as well as their fragmentation and elution orders (retention time) with previously published values and available standards. 

Compared to the other classes of quantified compounds, the most significant decrease was observed in HCA. This variation was expected due to the variation in the constituents and phenolic types among the different analyzed berries. These data correspond with similar conclusions of previous experiments reported regarding polyphenols antioxidant activity and their thermal degradation [43]. Moreover any processing of berries (especially thermal) and also storage were proved to be responsible for the significant losses of polyphenols. In a recent study, processing methods had insignificant effects on blueberry ellagitannins, but juice processing of berries resulted in total ellagitannin losses of about 70–82% [44]. Same author reported that storage at 25 degrees C of all processed products resulted in dramatic losses in monomeric anthocyanins with as much as 75% losses of anthocyanins throughout storage [45]. Another study, reported that thermal processes blackberries, showed a decreased content of anthocyanins (cyanidin-3-glucoside (by 52%) and cyanidin-3-malonyl glucoside (64%) respectively). They also reported that anthocyanins continue to decline during storage, especially when temperatures were high [46]. The obtained values for each type of berry of processed product are not easy to compare with literature available data due to the source of the berries or fruit. Their initial amount of polyphenols may vary with cultivation type, variety or climatic condition. 

For the in vitro test we have obtained promising results which can be compared with available published data. Many studies have proved that anthocyanins present beneficial effects for human health [47]. Because of their physiological activities, the consumption of anthocyanins may play a significant role in preventing lifestyle-related diseases such as cancer, diabetes, cardiovascular and neurological disease. However, the exact roles of the anthocyanins in human health maintenance versus other phytochemicals in a complex mixture from a fruit extract or whole food have not been completely sorted out. In vitro studies have shown various beneficial effects of anthocyanins regarding human health, but without doubt, in vivo, epidemiological and clinical trials would be more accurate. However, to the best of our knowledge, no report exists on the effect of processing on the phenolic compounds content of homemade jams from chokeberry, elderberry, blackcurrant or blackthorn.

## 4. Materials and Methods

All solvents, reagents, and standards used to perform the experiments were of analytical grade and purchased from Sigma-Aldrich (Darmstadt, Germany) The anthocyanin standards cyanidin-3-*O*-glucoside chloride, pelargonidin-3-*O*-glucoside chloride, cyanidin-3-*O*-galactoside (purity 90%), cyanidin-3-*O*-arabinoside (purity 97%), cyanidin-3-*O*-glucoside (purity 95%), and cyanidin (purity 95%) were purchased from Polyphenols AS (Sandnes, Norway). Chlorogenic acid, caffeic acid, quercetin-3-*O*-rutinoside, quercetin-3-*O*-glucoside, ellagic acid, and myricetin were also purchased from Sigma-Aldrich (Darmstadt, Germany). 

### 4.1. Sampling Procedure

Berry fruits (chokeberry, elderberry, blackcurrant, and blackthorn) were purchased from local farmers near Cluj-Napoca, Romania. Immediately after harvesting, the berries were frozen at −18 °C for future analyses. Basically, a common and simple jam-making procedure was followed in this study. Jams were prepared from berries using 250 g of sugar and 500 g of chopped berries, this being the most common ratio found in homemade jam products. The obtained mixture was heated in a gas stove (85 °C) for 15 min each day over 3 consecutive days. After boiling on the 3rd day, the jams were allowed to cool down to room temperature before being placed in glass jars (45 g). The obtained jams were analyzed immediately after preparation, and the remaining jams were divided into four batches and stored in the dark at 4 °C. Samples were analyzed after 1, 3, 6, and 9 months of storage. 

### 4.2. Extraction of Anthocyanin and Non-Anthocyanin Phenolics from Fresh Berries and Berry Jam

For the extraction of polyphenols, 5 g of fresh berries of each type were ground using an ultraturrax (Miccra D-9 KT Digitronic, Heitersheim, Germany). Additionally, the same amount of berry jams was homogenized and weighed, followed by the addition of 10 mL of methanol containing hydrochloric acid (0.3% *v*/*v*). The obtained mixtures were sonicated for 20 min in the dark and then centrifugated at 5000 rpm for 5 min. The supernatant was collected, and the extraction process was repeated until the samples were colorless. The extracts obtained for each sample were concentrated at 35 °C under reduced pressure (Rotavapor R-124, Buchi, Flawil, Switzerland) and then filtered through a 0.45 μm Millipore filter. All the sample preparation steps were carried out in subdued light and under controlled conditions. 

### 4.3. RP-HPLC-PDA Identification and Quantification of Phenolic Compounds

HPLC analysis was performed on a Shimadzu (Kyoto, Japan) system equipped with a binary pump delivery system (model LC-20 AT Prominence), a degasser (model DGU-20 A3 Prominence), a UV–VIS diode array detector (model SPD-M20), and a Luna C-18 column (film thickness, 5 μm; 25 cm, 4.6 mm) (Phenomenex, Torrance, CA, USA). The mobile phases were formic acid (4.5%) in double-distilled water (solvent A) and acetonitrile (solvent B). The gradient elution system was as follows: 10% B, 0–9 min; 12% B, 9–17 min; 25% B, 17–30 min; 90% B, 30–50 min; and 10% B, 50–55 min. The flow rate was 0.8 mL/min, and all analyses were performed at 35°C. Identification and peak assignments were conducted based on their retention times UV-VIS spectra, standards and available literature. The chromatograms were monitored at 340 and 520 nm. Anthocyanin quantification was conducted using a cyanidin-3-*O*-galactoside standard curve while for flavanol and phenolic acid was assessed using a rutin or chlorogenic acid standard curve. 

### 4.4. HPLC-PDA/-ESI-MS Identification and Quantification of Phenolic Compounds

To confirm the identified compounds, an ESI-MS analysis was also conducted. The ESI-MS analysis was performed using an Agilent 1200 system equipped with a binary pump delivery system (LC-20 AT, Prominence), a degasser (DGU-20 A3, Prominence), a diode array SPD-M20 A UV–VIS detector (DAD), and an Eclipse XDB C18 column (4 μm, 4.6 × 150 mm) was used. The mobile phases used the following solvents: (A) bidistilled water and 0.1% acetic acid/acetonitrile (99/1 *v*/*v*), and (B) acetonitrile and acetic acid 0.1%. The gradient elution system conditions were as follows: 0–2 min, isocratic with 5% (*v*/*v*) eluent B; 2–18 min, linear gradient from 5% to 40% (*v*/*v*) eluent B; 18–20 min, linear gradient from 40% to 90% (*v*/*v*) eluent B; 20–24 min, isocratic on 90% (*v*/*v*) eluent B; 24–25 min, linear gradient from 90% to 5% (*v*/*v*) eluent B; 25–30 min, isocratic on 5% (*v*/*v*) eluent B. The flow rate was 0.5 mL/min, and the column temperature was maintained at 25° C. The chromatograms were monitored at 280 and 340 nm, respectively. The identification of compounds was conducted based on their retention times, UV-VIS spectra, standards (chlorogenic acid, caffeic acid, quercetin-rutinoside, quercetin-glucoside, ellagic acid, and myricetin, all purchased from Sigma-Aldrich, and published data. The mass spectrometric data were obtained using a single quadrupole 6110 mass spectrometer (Agilent Technologies, Chelmsford, MA, USA) equipped with an ESI probe with scanning range between 280 to 1000 *m*/*z*. The measurements were performed in the positive mode, with an ion spray voltage of 3000 V and a capillary temperature of 350 °C. 

### 4.5. Cell Culture

The metastatic B16-F10 murine melanoma cell line was purchased from ATCC (Rockville, MD, USA) and grown under standard conditions. More specifically the cells were cultivated in DMEM (Dulbecco’s Modified Eagle Medium) medium containing 4.5 g/L glucose, 10% FBS supplemented with 2 mM glutamine, 1% penicillin, and streptomycin. The non-tumor model (HFL-1 human fetal lung fibroblast cell line, ATCC) was cultivated in a F-12K (Kaighn’s Modification of Ham’s F-12 Medium) medium containing 10% FBS and 1% penicillin/streptomycin. Both cell lines were maintained under standard conditions at 37 °C, 5% CO_2_ and 95% relative humidity.

### 4.6. Analysis of Cell Proliferation

For proliferation analysis, both cell lines were plated at a density of 8 × 10^3^ cells/well in a 96-well microplate and cultured in complete medium for 24 h. The medium was then replaced with a complete medium containing, or not containing rich polyphenolic extracts (RPE) at various concentrations (0–100 µg/mL) for 24 h at 37 °C with 5% CO_2_. The RPE stock solution was prepared with a complete medium containing 0.3% DMSO. The treatment was applied for 24 h at 37 °C with 5% CO_2_. To assess the cell viability after treatment with RPE we used a standard procedure. Briefly, the cell culture medium was removed and freshly prepared MTT reagent (0.5 mg/mL) was added to each well. After 2 h of incubation at 37 °C, the MTT solution was carefully removed, and DMSO was added in order to dissolve the formazan crystals that had formed in the mitochondria. The solubilized formazan formed in the viable cells was measured at 550 and 630 nm (for the sample and background, respectively) using the microplate reader, HT BioTek Synergy (BioTek Instruments, Winooski, VT, USA). The results are expressed as the survival percentage with respect to an untreated control. The control cells were assessed to be 100% viable. 

### 4.7. Statistical Analysis

Data are expressed as the mean ± standard error of mean (SEM) of three analyses of each sample. Analysis of variance (ANOVA) and Dunnett’s multiple comparisons test were used to determine significant differences between values (*p* < 0.05).

## Figures and Tables

**Table 1:** Chromatographic, mass spectral characteristics and tentative identification of compounds compounds in berry jams by LC-PDA-ESI/MS.

Peak No.	R_t_ (min)	Parent Ion	Fragment Ion	MW	UV Spectra	Compound	Ref
**Chokeberry**							
1	13.42	181	163	180	323	Caffeic acid	[22,23,25]
2	14.79	465	303	464	355	Quercetin-3-*O*-galactoside	
3	15.42	611	303	610	354	Quercetin-3-*O*-rutinoside (Rutin)	
4	16.11	465	303	464	355	Quercetin-3-*O*-glucoside	
5	16.29	-	303	302.1	364	Ellagic acid	
6	21.90	-	303	302	369	Quercetin	
7	10.80	449	287	449	528	Cyanidin-3-*O*-galactoside	
8	11.57	419	287	419	517	Cyanidin-3-*O*-arabinoside	
9	12.58	419	287	454	519	Cyanidin-3-*O*-xyloside	
10	13.20	449	287	449	518	Cyanidin-3-*O*-glucoside	
11	14.31	-	287	287	528	Cyanidin	
**Blackthorn**							
1	13.14	181	163	180	319	Caffeic acid	[26]
2	14.47	355	181, 163	354	325	Neochlorogenic acid	
3	15.19	611	303	610	354	Quercetin-3-*O*-rutinoside (rutin)	
4	21.38	303		302	369	Quercetin	
5		449	287	449	528	Cyanidin-3-*O*-galactoside	
6	10.84	595	449, 287	595	516	Cyanidin-3-*O*-rutinoside	
7	11.96	609	463, 301	611	524	Peonidin-3-(*6*″-coumaroyl) glucoside	
**Elderberry**							
1	13.39	355	181, 163	354	352	Chlorogenic acid	[27]
2	15.44	611	303	610	354	Quercetin-3-*O*-rutinoside	
3	16.14	465	303	464	355	Quercetin-3-*O*-glucoside	
4	21.38	-	303	302	369	Quercetin	
5	10.94	581	449, 287	616	518	Cyanidin-3-*O*-sambubioside	
6	14.29	449,	287	449	518	Cyanidin-3-*O*-glucoside	
**Blackcurrant**							
1	11.16	301	139	300	252	4-Hydroxybenzoic acid-4-*O*-glucoside	[12,28,29]
2	13.59	343	181, 163	343	321	Caffeic acid-4-*O*-glucoside	
3	13.97	595	287	594	346	Kaempferol-3-*O*-rutinoside	
4	14.59	627	319	626	355	Myricetin-3-*O*-rutinoside	
5	15.04	465	319	646	372	Myricetin-3-*O*-rhamnoside	
6	15.66	611	303	610	354	Quercetin-3-*O*-rutinoside	
7	16.37	465	303	464	355	Quercetin-3-*O*-glucoside	
8	16.70	357	195	194	316	Ferulic acid-4-*O*-glucoside	
9	17.47	449	287	448	346	Kaempferol-3-*O*-galactoside	
10	17.84	567	319	566	355	Myricetin-3-*O*-(*6*″-malonyl-glucoside)	
11	19.25	319		300	255	Hydroxybenzoic acid-4	
12	20.25	551	303	550	358	Quercetin-3-*O*-(*6*″-malonyl-glucoside)	
13	21.99	303		302	369	Quercetin	
14	22.53	449	287	448	346	Kaempferol-3-*O*-glucoside	
15	22.98	287		286	365	Kaempferol	
16	10.12	465	*303*	465	524	Delphinidin-3-*O*-glucoside	
17	10.57			611	528	Delphinidin-3-*O*-rutinoside	
18	11.38	595	*287*	449	518	Cyanidin-3-*O*-glucoside	
19	14.38	449	287	287	514	Cyanidin	
20	15.64	303		303	520	Delphinidin	

## Abbreviations

3-(4,5-Dimethylthiazol-2-yl)-2,5-Diphenyltetrazolium Bromide	MTT
anthocyanins	ANT
Flavonols	FLA
Foetal bovin serum	FBS
Hydroxybenzoic acids	HBA
Hydroxycinnamic acids	HCA
Metastatic murine melanoma cell line	B16-F10
Normal fibroblast cell line	HFL-1
Rich polyphenolic extracts	RPE
